# A “Wastes-Treat-Wastes” Technology: Role and Potential of Spent Fluid Catalytic Cracking Catalysts Assisted Pyrolysis of Discarded Car Tires

**DOI:** 10.3390/polym13162732

**Published:** 2021-08-15

**Authors:** Baishun Zhao, Chuansheng Wang, Huiguang Bian

**Affiliations:** 1 College of Electromechanical Engineering, Qingdao University of Science and Technology, Qingdao 266061, China; bianhuiguang@163.com; 2 State Key Laboratory of High Performance Complex Manufacturing and School of Mechanical and Electrical Engineering, Central South University, Changsha 410083, China

**Keywords:** spent catalyst, pyrolysis, waste tires, thermogravimetry

## Abstract

Spent fluid catalytic cracking catalysts (FCC catalysts) produced by the petrochemical industry are considered to be environmentally hazardous waste, and precious metals and heavy metals deposited on the surface make them difficult to treat. Even so, these catalysts retain some of their activity. The pyrolysis of waste tires is considered to be one of the most effective ways to solve the fossil fuel resource crisis, and this study attempts to catalyze the pyrolysis of waste tires using spent catalysts to increase the value of both types of waste. FCC catalysts reduced the activation energy (*E*) of waste tire pyrolysis. When the catalyst dosage was 30 wt.%, the *E* of tread rubber decreased from 238.87 kJ/mol to 181.24 kJ/mol, which was a 19.94% reduction. The *E* of the inner liner decreased from 288.03 kJ/mol to 209.12 kJ/mol, a 27.4% reduction. The spent catalyst was more effective in reducing the *E* and solid yield of the inner liner made of synthetic rubber. It should be emphasized that an appropriate increase in the heating rate can fully exert the selectivity of the catalyst. The catalyst could also be effectively used twice, and the optimum ratio of catalyst/waste tires was about 1/4.5. Compared with specially prepared catalysts, it is more cost-effective to use such wastes as a catalyst for waste tire pyrolysis.

## 1. Introduction

The use of fossil fuels and environmental protection are a popular topic of discussion [1], and mainly focuses on the effective use of renewable resources and the recycling of waste. Waste tires are a common form of waste, which has been described as “a kind of resource misplaced” [2]. Natural rubber, the main component of tires, is an important renewable hydrocarbon and releases significant amounts of heat when burned. Waste tires can be used in this manner, but if they are directly used as a fuel, they will generate significant amounts of acidic gas and dust, leading to environmental pollution [3]. Other treatment methods, such as landfills or stacking, have their own drawbacks, such as occupying a large area of land, becoming a hotbed for the growth of mosquitoes and bacteria, and releasing unpleasant gases. Therefore, value-added and cleaning treatment of waste tires has been plagued by major challenges.

Nowadays, pyrolysis is considered a part of the complex biomass and waste processing concept [4]. Pyrolysis is a thermochemical treatment method that has unique advantages in the final product, treatment process, and environmental protection, and has attracted the attention of many researchers [5]. When tires are pyrolyzed, polymer chains are degraded by heating under an inert atmosphere to generate small molecular compounds [6]. The main liquid products are potential substitutes for fossil fuels, and pyrolysis also has optimistic application prospects for the treatment of coal, biomass, and solid waste. For example, the pyrolysis of plastic casings of televisions and computers and other plastic waste products has been studied [7,8]. Liquid hydrocarbon biofuels can also be obtained from microalgae or waste cooking oil by catalytic pyrolysis [9,10], and natural macromolecular materials such as cooking oil, rubber seed, and Chlorella can also produce useful pyrolysis products [11,12,13,14].

Waste tires processed from polymers can be used as sources of hydrogen and light hydrocarbons due to their chemical compositions. However, oil products are not suitable for direct use as gasoline because they are highly viscous, have a high ash content, and also contain complex polycyclic aromatic hydrocarbons and sulfur or nitrogen compounds [15]. Therefore, the use of catalysts to remove heteroatoms (N and S) from pyrolysis product oils, reduce their viscosity, and remove light hydrocarbon species has become increasingly popular.

Zeolite-based catalysts were the first catalysts introduced for the pyrolysis of waste tires, including HMOR, HZSM-5, and MCM-41 [16]. The good cracking characteristics of zeolites can transfer the distribution range of polar aromatics from heavy fraction in the range of gas oil to light fraction in the range of kerosene. At the same time, the S content of pyrolysis oil can be reduced by using highly acidic zeolites [16]. Although zeolites promote the production of polyaromatic hydrocarbons (PAHs) in pyrolysis oil, metal-loaded zeolites can increase the quality and quantity of pyrolysis oil. Such catalysts can simultaneously catalyze a variety of reactions, including hydrogenation, dehydrogenation, ring-opening, cracking, and aromatization [17]. After Ilkilic et al. found that noble metals (Pt, Ru, Re, and Rh) can be used as the supporting metal of zeolite, Elbaba et al. found that transition metals (Fe, Ni, and Cu) also have this function [18,19].

Yuwapornpanit et al. found that zeolites (Hβ, HY, and HMOR) loaded with 5 wt.% Cu could reduce the sulfur content of pyrolysis oil, but benzene and ethylbenzene were found in the final pyrolysis products [20]. Muenpol et al. found that zeolites (HMOR, Hβ, HZSM-5, and KL) loaded with 5 wt.% Fe promoted an increase in the light fraction in pyrolysis oil by improving the cracking capacity, and also reduced the amount of sulfur, double aromatics and PAHs [21]. Kordoghli et al. found that when MgO was used as a catalyst, the recovery yield of hydrogen was increased from 14% to 32% by volume [22]. The use of catalytic pyrolysis to treat non-biodegradable materials and obtain clean energy has shown considerable potential [23,24]. Various catalysts such as K_2_O/Mg-MCM-41 [25], CaO, and HZSM-5 [26], Zr and Mo modified MCM-41 [27], ZrO_2_/SBA-15 [9,28] have been used to produce liquid hydrocarbons and enhance the quality of pyrolysis products. Ni has also proven to be useful for the pyrolysis of waste tires and during the production of hydrogen [29,30,31,32]. Wang et al. found that the addition of CeO_2_ helped improve the catalytic activity and reduce the carbon deposition rate when studying CeO_2_ and Al_2_O_3_ loaded with Ni [33]. Ultimately, it is the high cost that limits the application of these catalysts in industrial processes, and the reasonable use of such catalysts has not been described in detail.

Sulfur and other impurities can be removed from crude oil if a large amount of solid metal is applied during the hydrotreatment stage of the refining process [34]. However, in this reaction, the deposition of rare metals (Ni, V, etc.) and coke reduces the catalytic activity, and the amount of the spent fluid catalytic cracking (FCC) catalyst reaches about 170,000–200,000 tons per year [35]. Spent FCC catalysts have been listed as hazardous wastes by many countries because of the presence of toxic metals [36]. Interestingly, these spent catalysts, which contain metals such as Ni, V, Co, Al, and Fe, are used as raw materials to catalyze the pyrolysis of waste tires. However, there is very little research on the application of these catalysts for the catalytic treatment of waste tires.

In the relevant literature, the types of catalysts and corresponding pyrolysis products have been studied intensely, but the cost of catalysts, recycling, and the effects on thermal kinetics have rarely been studied. Therefore, the focus of this study is to determine the feasibility of using spent FCC catalyst to catalyze the pyrolysis of waste tires by analyzing its components and its impact on thermal kinetics. The rationality and utilization value of spent catalysts for the catalytic pyrolysis of waste tires were analyzed using thermogravimetric analysis. Then, through the above reaction parameters, the optimal input ratio of spent catalyst in the catalytic pyrolysis process and its theoretical significance were determined. This helped determine the evolution of pyrolysis and depict the process in terms of mathematical and physical models. At the same time, the research also provides a way to deal with the two identified wastes and increase their recycling value.

## 2. Materials and Methods

### 2.1. Materials

The raw materials used in this experiment were car tires provided by TIRE TECHNOLOGY ALLIANCE (TTA, Qingdao, China). The company also provided the corresponding formulae of the tire inner liner and tread rubber, which are shown in Table 1. The most basic functions of tread rubber are wear resistance and skid resistance, while the inner liner needs to be airtight to store compressed gas in order to ensure normal tire operation. Therefore, the different functions of tire components determine the differences between the formulas of the various materials, which also determine the different pyrolysis behaviors [37,38]. This tire was chosen because tires with silica as the main filler are representative, it is viewed as a green tire and it is widely used all over the world. [39,40]. Before the experiment, a sufficient amount of the inner liner and tread rubber were taken and then crushed into particles of about 1 mm. Finally, the particles were stored in a drying oven at 80 °C for 6 h to remove moisture.

Sinopec Jinan Branch (Jinan, China) provided the spent FCC catalyst with a micro-activity of approximately 65%, which complies with the Chinese test standard NB/SH/T 0952-2017. The spent catalyst is a gray powder with a subtle pungent odor. Before use, a sufficient amount of powder was stored in a drying oven at 80 °C for 6 h to remove moisture. Table 2 shows its components.

### 2.2. Thermogravimetric Experiments

This study was carried out using a thermogravimetric analyzer (TG 209 F3 Tarsus, NETZSCH, Germany; temperature range: 0 °C to 1100 °C; weight range: 0 to 2000 mg). Although this technique is necessary for conducting comprehensive thermal analysis, the thermogravimetric analysis results are very similar to the actual pyrolysis process of the tire, regardless of whether catalysts are spent or not [41]. The thermogravimetric tests were carried out using high-purity nitrogen as purge gas (50 mL/min) and shielding gas (40 mL/min). The heating rates (10, 15, 20, 25 K/min) and temperature ranges (40 °C to 550 °C) used during the test are consistent with the conditions used for slow solid pyrolysis in actual reaction operations [42,43,44]. The weight of the raw materials was about 15 mg, as measured by the thermogravimetric analyzer. The weight of the catalyst used is 1.5 mg, 3 mg, 4.5 mg and 6 mg (10 wt.%, 20 wt.%, 30 wt.% and 40 wt.% of the pyrolysis material, respectively). Before experiment, the crushed tire samples and catalyst powder were mixed into the test crucible and shaken sufficiently. Finally, the optimal input ratio was determined by fitting the change in the activation energy under different catalyst usage conditions.

### 2.3. Thermal Analysis Kinetics

The multiple scanning rate method refers to a kinetic analysis method for multiple TG curves obtained at different heating rates. Among them, the conversion methods such as the Friedman method, KAS method, and FWO method are representative. This method can achieve separate solutions of three dynamic parameters. This allows a reliable activation energy *E* to be solved independently without involving the kinetic model function [45].

Pyrolysis of the waste silica-filled tire follows the non-isothermal heterogeneous reaction kinetic equation. In general, Equation (1) is used to describe the thermal decomposition kinetics of solids:

(1) $$\frac{d\alpha }{dT}=\frac{A}{\beta }\;\exp\;\left(-\frac{E}{RT}\right)\;f\left(\alpha \right)$$

(2) $$\alpha =\frac{m_{0}-m}{m_{0}-m_{\infty }}$$

where α is the pyrolysis conversion rate, *T* is the pyrolysis temperature (K), β is the heating rate (K/min), *A*, *E*, and *R* are pre-exponential factors (s^−1^), activation energy (J/mol), and general gas constant (J/(mol•K)), respectively, f(α) is a reaction mechanism function that controls the reaction process, and *m*_0_, *m*, and *m*_∞_ are the initial mass, transient mass, and final mass (mg) of the sample during pyrolysis, respectively.

The Starink equation (Equation (3)) in the equal conversion method is considered to be more accurate than the FWO method and the KAS method when solving the activation energy [46,47]. Therefore, the Starink method was used in this study.

(3) $$\ln\left(\frac{\beta }{T^{1.8}}\right)=-\frac{BE}{RT}+constant$$

where *B* = 1.0037, and *R* is the universal gas constant (8.314 J/(mol•K)). To start, the different heating rates β and temperature *T* at the same conversion rate α were substituted into the above equation. Furthermore, the activation energy of each stage was characterized by the slope of the straight line in the $\ln\left(\beta /T^{1.8}\right)$-*B/RT* diagram. The advantage of this method is that the calculation error of the kinetic parameters due to the assumption f(α) can be excluded.

## 3. Results and Discussion

### 3.1. Thermogravimetric Analysis

**Figure 1:**
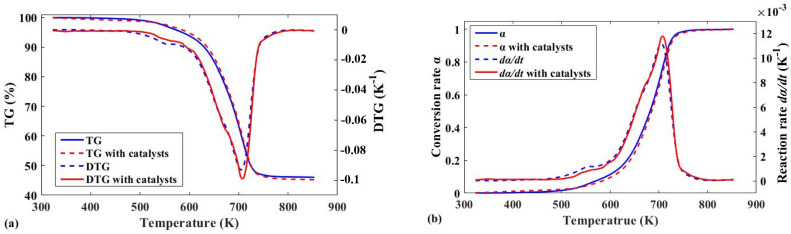

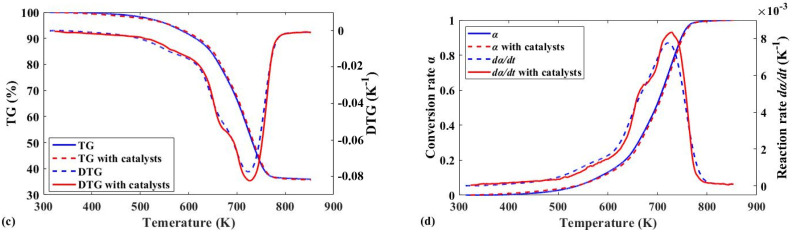
(**a**) TG and DTG curves of inner liner using spent catalysts (4.65 mg) or not; (**b**) Conversion rate and reaction rate curves of inner liner using spent catalysts or not; (**c**) TG and DTG curves of tread rubber using spent catalysts or not; (**d**) Conversion rate and reaction rate curves of tread rubber using spent catalysts (4.42 mg) or not. (*β* = 25 ^o^C min, Temperature range: 40–600 ^o^C, Shielding gas and Rate: N2, 40 mL min^−1^ Purge gas and Rate: N_2_, 50 mL min^−1^, Crucible: AL_2_O_3_).

Figure 1a shows the TG and DTG curves of the inner-liner pyrolyzed with and without catalyst at a heating rate of 25 K/min. First, the TG curve generally did not drastically change, but there were some differences. The addition of catalyst reduced the amount of final solid product by nearly 1%, which means that the presence of catalyst promoted the conversion of solid products to gas products, which improved the gas recovery rate. Of course, the significance of the conclusion is limited, but almost all experimental results show the same trend. As mentioned in the introduction, some metals (Ni, V, Fe) can improve the gas/solids ratio [17]. At the same time, the presence of V inhibits the formation of coke [48]. Some useful metals (Ni, V, Fe) for the pyrolysis of waste tires were deposited on the surface of these catalysts after the petroleum refining process. Table 2 shows that many components beneficial to pyrolysis are present in the spent catalyst, but are present in low amounts, which limits the effectiveness of the catalyst. In addition, the reduction in the amount of coke can improve the conversion rate, and also increase the heat transfer efficiency and extend the cleaning cycle of equipment. By comparing the TG curves in Figure 1a,c, it was found that the response of the inner liner to the final solid product recovery appeared to be more obvious. In fact, from the comparison of the formulae in Table 1 and Table 2, the main material constituting the inner liner was synthetic rubber, which is manufactured from petroleum products [49]. Therefore, the spent FCC catalyst better reduced the coking of the inner liner. Table 3 gives more detailed data for some of the thermogravimetric experiments.

**Figure 2:**
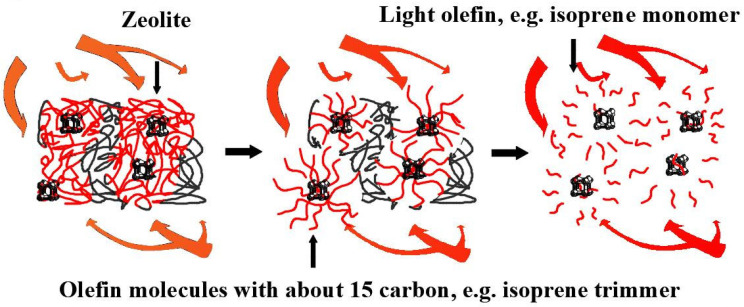
Schematic diagram of zeolite function.

Secondly, the data in Table 3 show that in the presence of a catalyst, when the pyrolysis start temperature moves to the high-temperature region, the end temperature hardly changed. This reflects that the spent catalyst helps increase the pyrolysis efficiency, which was verified by comparing the minimum of the DTG curve or the maximum conversion rate in Figure 1b,d. In fact, the introduction of spent catalyst did not increase the reaction temperature of pyrolysis or reduce its apparent activation energy, as demonstrated in the next section. The DTG curves in Figure 1a,c explain the cause of this phenomenon. Compared with catalytic pyrolysis, the first weight loss peak of the conventional pyrolysis reaction was prominent, indicating a higher weight loss rate and greater weight loss. The initial pyrolysis reaction stage mainly consisted of the pyrolysis of a small portion of the organic molecular backbone. The main reason for the transfer of the weightlessness reaction zone to the high-temperature zone is the zeolite in the spent catalyst. The tread rubber formulae listed in Table 1 shows that the main component is NR, which will pyrolyze to produce an isoprene trimer during the initial reaction stage [50]. Zeolite (aluminosilicate) is a porous molecular sieve. Subsequently, the molecular group is adsorbed by the porous molecular sieve, preventing it from escaping with the gas. Therefore, the spent catalyst slowed the weight loss rate and reduced weight loss at this stage. Then, as the pyrolysis temperature gradually increased, some small-molecule aggregates underwent a secondary pyrolysis reaction when there was sufficient thermal energy to produce smaller molecular weight substances, such as isoprene monomer. These small molecular products were able to eliminate the adsorption of molecular sieves and thus constitute pyrolysis products in the high-temperature region. This process is shown in Figure 2. Therefore, from the TG curve, the pyrolysis temperature seems to be high, but the characteristics of the catalyst cause this phenomenon. Table 3 shows that this phenomenon became more apparent as the heating rate increased, which indicates that an appropriate increase in the heating rate can fully exert the selection effect of the catalyst. Furthermore, the quality of the pyrolysis oil was improved by reducing the diversification of the oil molecules. Figure 1b,d show that the spent catalyst has the same effect in reducing the amount of multimer in the gas product. In order to verify the above catalyst mechanism, waste tire pyrolysis gas and catalytic pyrolysis gas were collected and analyzed with a gas chromatography mass spectrometer (Agilent 7890-975c GC-MS, Beijing, China). Pyrolysis gas was collected from laboratory-made pilot equipment, which is similar to general pyrolysis equipment, including heating burner, pyrolysis rotary kiln, two-stage pyrolysis oil condensing unit, tail gas treatment unit and other auxiliary units. As there were multifarious components, only part of the data (≥1%) is listed in Table 4. GC-MS data clearly shows that the composition of catalytic pyrolysis gas tends to be homogenized, and its main component is C_4_H_8_ (1-Propene, 2-methyl-), accounting for 65.59%. The content of hydrocarbon with 4 or less carbon atoms reached 83.7%, which was 32.28% higher than that of pyrolysis gas without catalyst (51.42%). Therefore, the catalyst can improve the uniformity of pyrolysis gas, which further improves its utilization possibility.

### 3.2. Kinetics Analysis

**Figure 3:**
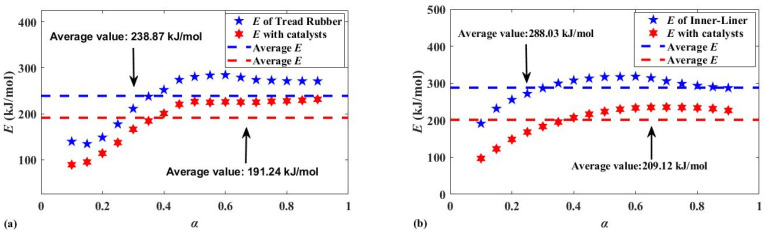
(**a**) Comparison of activation energy of tread rubber; (**b**) Comparison of activation energy of inner liner.

Figure 3a,b show the effect of the spent catalyst on the activation energy of the two compounds at a catalyst loading of 30 wt.%. The spent catalyst had an obvious effect on *E* during various stages of the pyrolysis reaction, which can be seen from the two activation energy comparison charts. By comparing them, it can be seen that spent catalysts significantly reduced the apparent activation energy, which means less energy input was required during pyrolysis. Importantly, this explains why the temperature at which the pyrolysis reaction occurs was lowered. In addition, the *E* of tread rubber decreased from 238.87 kJ/mol to 181.24 kJ/mol when the catalyst was used, which is a decrease of 19.94%. *E* decreased from 288.03 kJ/mol to 209.12 kJ/mol, when the inner liner was pyrolyzed, representing a decrease of 27.4%. It can be seen that the application of the spent FCC catalyst during the pyrolysis of waste tires can help lower the activation energy. Combined with the formula of the two compounds listed in Table 1 and Table 2, this spent catalyst will have a more remarkable effect when reducing the *E* of the inner liner made from synthetic rubber.

**Figure 4:**
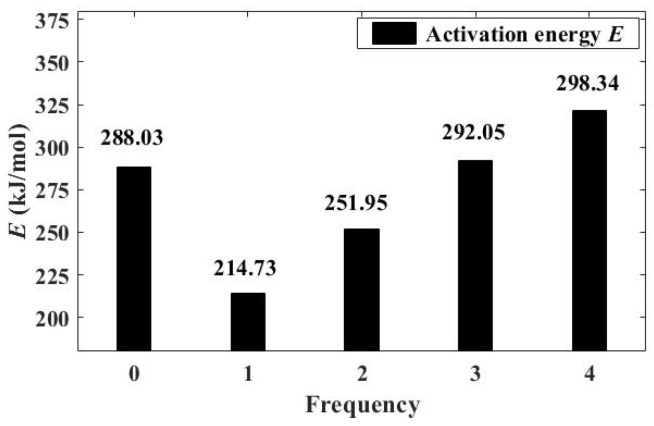
Activation energy of inner liner at different catalysts (20 wt.%) usage times.

Next, the recyclability of this type of catalyst after catalyzing the pyrolysis of waste tires was studied. As mentioned above, the inner liner is more sensitive to the presence of a catalyst, so it was used as the research object. The pyrolyzed rubber was uniformly mixed with the catalyst prior to thermogravimetric tests. After pyrolysis, the solid residue is mixed with the catalyst and cannot be separated, which is consistent with the current industrial treatment of waste tire pyrolysis. Therefore, when calculating the *E* of catalyst used for the second time, the pyrolysis of raw material was directly added to the crucible and mixed uniformly. After four replicates, the resulting *E* of the pyrolysis reaction is shown in Figure 4. The activation energy of each period listed in Figure 4 is the arithmetic average value calculated after multiple scans, as described in Section 2.3. In this respect, the spent catalyst lost some of its activity after each tire pyrolysis. Figure 4 shows that compared with its first use, *E* sharply decreased from 25.45% to 12.53% during its second use. After being used twice, the catalyst shows almost no effect on *E*. Even with the increase of reaction times, the *E* of the reaction still tended to increase because each pyrolysis produced 35–45 wt.% of solid products. This is because the surface of the catalyst continuously accumulates impurities such as pyrolytic carbon black and dust, which will gradually block the pores of the catalyst, causing it to eventually lose its activity [51]. According to the test results, the specific surface area of the catalyst decreased about 19.37% before and after the experiment, as shown in Table 5. Meanwhile a large amount of pyrolytic solid powder reduces the effective contact rate of the catalyst with the reactants. Furthermore, due to the accumulation of a large amount of carbon black, the solid base of the spent catalyst formed a carbanion by the electron-accepting ligand is separated by them, which further deactivated the catalyst, because solid bases and free olefins do not interact effectively. Therefore, the spent catalyst could only be used twice during the catalytic pyrolysis of the waste tire without any treatment. Even when it was used for a second time, its catalytic activity decreased dramatically.

### 3.3. Analysis of Catalyst Dosage

The effects of catalyst dosage are often neglected by researchers, and the catalyst usage ratio is generally used as an empirical value or determined according to the final products. Based on the ratio and production of gas and liquid byproducts, Kordoghli et al. determined a very low catalyst/waste ratio (1/30) [22]. Elbaba et al. used two-stage gas pyrolysis to produce hydrogen gas under a catalyst/waste tire ratio of 0.5, a pyrolysis temperature of 500 °C, and a gasification temperature of 800 °C [18]. Catalyst/waste tire ratios of 1/4, 1/2 and 1/1 were used in the second catalytic reactor [52,53]. Determining the catalyst dosage according to the distribution of products tends to ignore an important issue, which is the economic feasibility of the process. If the income generated from the final product is much lower than the input cost of the catalyst, then such a catalyst would have limited industrial significance. For example, in order to obtain hydrogen, some precious metals such as Pt, Pd, Rh, and Ru are used as catalysts during the pyrolysis process of waste tires [54,55]. Due to their high costs, they cannot be applied in large quantities. This study attempts to analyze the catalyst usage ratio from the perspective of activation energy to identify a low-cost, high-profit catalyst. The purpose is to improve the pyrolysis efficiency of waste tires, reduce the pyrolysis input energy, and maximize the utility and value of spent catalysts.

**Figure 5:**
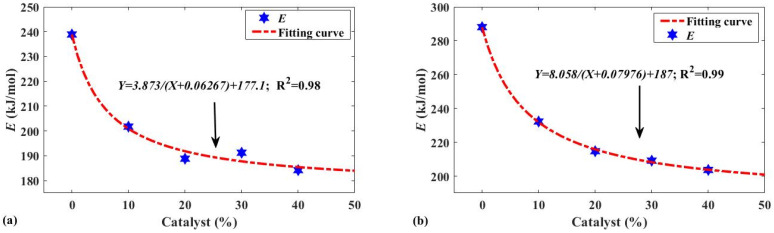
(**a**) Activation energy fitting results of tread rubber at different catalyst dosages; (**b**) Activation energy fitting results of inner liner at different catalyst dosages.

In order to better determine the relationship between catalyst dosage and activation energy, the *E* was determined at catalyst dosages of 10%, 20%, 30%, 40% of the mass of the compound. Figure 5a,b show the changes in the activation energy of tread rubber and inner liner as the catalyst consumption increased, respectively. Detailed data are listed in Table 6. The figure clearly shows that the rate of *E* decreases significantly with an increasing catalyst dosage. In other words, the maximum catalytic effect of the catalyst gradually stabilizes, and the catalyst dosage gradually becomes excessive. In addition, there seems to be an inverse proportional function between the two, which is derived from the trend of *E* in Figure 5. Fitting the data with the inverse proportional function model (4) provides a better understanding of the relationship between them and helps determine the optimal catalyst dosage. The linear correlation coefficient of the fitted curve in Figure 5 is quite high, indicating that the optimum catalyst dosage should be the highest limit in combination with the input cost of the catalyst and *E*. Choosing a reasonable ratio of catalyst use can not only fully utilize its catalytic effect but also effectively control costs. The catalytic activity depends on the material used, as shown by comparing Figure 5a,b, and the optimum ratio of catalyst used will vary somewhat depending on the material. From the perspective of activation energy, the optimum dosage of catalyst used in the catalytic pyrolysis of the tread rubber was from 20 wt.% to 30 wt.%. In contrast, when the raw material was the inner liner, the optimum dosage of catalyst was 30 wt.% to 40 wt.%, which is a better choice. The difference between them was not dramatic. Therefore, when the entire waste tire is pyrolyzed, the catalyst amount should be about 30% of the weight of tires. In addition, the optimum ratio of spent catalyst/waste tires was about 1/4.5.

(4) $$Y=\frac{a}{\left(x+b\right)}+c$$

### 3.4. Catalyst Residual Value

In the previous section, the catalyst proved to be effective for two cycles, so it is worth studying a means to increase the effective use of the catalyst. In this regard, the literature indicates that burning or acid cleaning can restore some catalytic activity [56,57]. Although acid cleaning is a viable option, it does not apply here because, at the end of pyrolysis, a solid mixture (30% catalyst and 40% solid product) of about 70 wt.% tire was produced. If these fine particles are washed with acid, the cost will increase due to the subsequent treatment process, and there is also the risk of secondary pollution. The spent catalyst was heated to 1000 °C in nitrogen as a comparative condition and then used to catalyze the pyrolysis of the inner liner. Since the solid product contains a large amount of carbon black, the catalyst which catalyzed the pyrolysis of waste tires cannot be heated in the air because a large amount of carbon black will undergo a vigorous redox reaction. This will result in the solid product being completely unrecyclable. The experimental results and detailed experimental conditions are listed in Table 7. By comparing the *E* of experiments 1–3 in Table 7, the spent catalyst which was heated before the catalytic pyrolysis of the waste tire lost its catalytic activity. This treatment did not improve the catalytic activity because the crystal lattice of the catalyst was basically destroyed at 700–800 °C. Since its physical structure was destroyed, it lost all catalytic effects [58]. In addition, some literature has reported that the presence of Fe and V (present in the spent catalyst) when heated to high temperatures can lead to the destruction of the zeolite structure [59]. Experiments 4 and 5 were comparison experiments in which the spent catalysts were used to catalyze the pyrolysis of waste tires. The difference was that the catalyst in experiment 5 was heated at a high temperature before the second catalysis. The results show that high-temperature heating nearly deactivated the catalyst. This is why the catalyst will degrade when it is used for the second time, causing it to have no activity when used for a third time. Thus, the spent FCC catalyst used for the pyrolysis of waste tires completely lost its recyclability and could not be further processed.

## 4. Conclusions

Spent FCC catalysts produced by the petrochemical industry still retain some activity, but precious metals and heavy metals deposited on their surface make give them considerable recycling value. At the same time, the pyrolysis of waste tires is one of the most effective ways to alleviate the stress of fossil fuels.

This study found that, like other catalysts, the spent catalyst significantly affected the composition and evolution of pyrolysis gases. It shifts the initial weight loss stage of pyrolysis from low temperature to high temperature, which makes some molecular chains undergo pyrolysis reaction again and produces smaller molecular groups. Furthermore, the composition of pyrolytic oil is more uniform and the quality is improved. From the perspective of thermal analysis kinetics, the spent catalyst reduced the activation energy of waste tire pyrolysis. When the dosage of catalyst was 30 wt.%, the activation energy of tread rubber decreased from 238.87 kJ/mol to 181.24 kJ/mol, a reduction of 19.94%. The activation energy of the inner liner decreased from 288.03 kJ/mol to 209.12 kJ/mol, which was a reduction of 27.4%. The spent catalyst more effectively reduced the activation energy and solid yield of the inner liner made of synthetic rubber. It should be emphasized that an appropriate increase in the heating rate can fully exert the selectivity of the catalyst. Finally, the experimental results showed that the catalyst could effectively be used twice, but the catalytic activity was reduced in its second use. The spent catalyst will lose all activity due to severe crystal lattice after being used twice. The optimum ratio of catalyst/waste tires was about 1/4.5. 

Future work will focus on how to separate spent catalysts and pyrolyzed solid products or find ways to treat solid mixtures.

## Figures and Tables

**Table 1:** Formulation of the test samples.

**Tread Rubber**	**Mixed Ingredients (PHR)**											
	SBR	NR		TSR20		Silica		V700		N234		Antilux
	15	105		20		70		13		18		3
	Si69	SAD		DPG		ZnO		S		CZ		Antioxidant 4020
	13	3		1		2		1.15		2		2
**Inner liner**	**Mixed ingredients (PHR)**											
	BR9000		SBR		TSR20		N234		TDAE		RD	
	15		15		70		45		3		1.5	
	SAD		ZnO		Antilux		6PPD		CBS		S	
	3		3.5		1.5		2		1.5		1.8	

Note: PHR—parts per hundreds of rubber, SBR—styrene butadiene rubber, NR—natural rubber, TSR20—technical standard natural rubber #20, Silica—silicon dioxide, V700—aromatic oils meeting EU standards, N234—carbon black, Antilux—ceresin wax, Si69—silane coupling agent, SAD—stearic acid, DPG—1,3-diphenylguanidine, CZ—N-cyclohexyl-2-benzothiazolylsulfenamide, Antioxidant 4020—N-(1,3-Dimethylbutyl)-N-Phenyl-p-phenylene Diamine, BR9000—butadiene rubber, TDAE—treated distillate aromatic extract, RD—Antioxidant RD, Antilux—ceresin wax, 6PPD—N-(1,3-dimethylbutyl)-N’-phenyl-p-phenylenediamine, CBS—N-cyclohexyl-2-benzothiazolylsulfenamide.

**Table 2:** X-ray fluorescence semiquantitative analysis of the spent FCC catalyst.

Component	Content (wt.%)	Component	Content (wt.%)
Na_2_O	0.226	CaO	0.7
MgO	0.42	TiO_2_	0.25
Al_2_O_3_	51.7	V_2_O_5_	0.246
SiO_2_	38.4	Fe_2_O_3_	1.12
P_2_O_5_	0.55	NiO	0.854
SO_3_	0.832	Sb_2_O_3_	0.245
K_2_O	0.252	La_2_O_3_	2.73

Note: loss on ignition—1.475%.

**Table 3:** Thermogravimetric results with spent catalysts or not.

Material	*β*/^o^C·min^−1^	W/mg	W_SFCC_/mg	T_e_/^o^C	T_f_/^o^C	(dα/dt)_max_/% K^−1^	T_max_/^o^C	W_f_/%
Inner liner	10	15.4	0	361.2	441.6	6.88	421.7	45.81
		15.7	4.55	360.1	441.0	6.84	421.9	44.83
	15	15.42	0	362.7	448.1	9.81	426.8	45.73
		15.22	4.62	363.2	448.6	10.59	424.1	44.79
	20	15.35	0	365.5	452.1	12.94	428.4	45.69
		15.7	4.62	368.3	453.0	13.33	433.2	45.00
	25	15.5	0	367.8	455.3	15.65	435.5	45.93
		15.21	4.65	370.9	457.1	16.24	435.4	45.00
Tread rubber	10	15.62	0	362.3	469.7	5.68	432.2	35.86
		15.62	4.56	363.7	469.9	5.62	433.1	35.19
	15	15.01	0	362.6	476.4	8.72	434.8	35.74
		15.06	4.46	369.5	477.3	8.73	443.0	35.17
	20	15.07	0	366.4	481.1	10.35	450.3	35.57
		15.49	4.72	371.9	482.8	11.12	444.3	35.24
	25	15.02	0	369.4	485.0	13.34	449.8	35.88
		15.46	4.42	374.2	486.5	15.41	456.1	35.65

Note; *β*—Heating rate, W—Weight, W_SFCC_—Weight of catalyst, T_e_—Temperature at which pyrolysis starts, T_f_—Temperature at which pyrolysis ends, (dα/dt)_max_—Maximum weightlessness rate, T_max_—Temperature at which weightlessness is fastest, W_f_—Residual solids.

**Table 4:** Pyrolysis gas components and content.

Experiment	No.	Matches Name	CAS Number	Molecular Formula	Proportion
Pyrolysis	1	1-Methylcyclopropene	003100-04-7	C_4_H_6_	48.07%
	2	1,3-Butadiene, 2-methyl-	000078-79-5	C_5_H_8_	19.34%
	3	Cyclopropane, 1,1-dimethyl-	001630-94-0	C_5_H_10_	14.08%
	4	1-Butene, 3-methyl-	000563-45-1	C_5_H_10_	4.29%
	5	Propene	000115-07-1	C_3_H_6_	2.04%
	6	Isobutane	000075-28-5	C_4_H_10_	1.31%
	7	Cyclopentene	000142-29-0	C_5_H_8_	1.13%
	8	2-Pentene, 4-methyl-, (Z)-	000691-38-3	C_6_H_12_	1.07%
		⋯	⋯	⋯	⋯
FCC catalytic pyrolysis	1	1-Propene, 2-methyl-	000115-11-7	C_4_H_8_	65.59%
	2	Propylene	000124-38-9	C_3_H_6_	18.11%
	3	Cyclopropane, 1,1-dimethyl-	001630-94-0	C_5_H_10_	3.70%
	4	2-Pentene, (E)-	000646-04-8	C_5_H_10_	2.45%
	5	1,4-Pentadiene	000591-93-5	C_5_H_8_	2.16%
	6	Isobutane	000075-28-5	C_4_H_10_	1.68%
		⋯	⋯	⋯	⋯

**Table 5:** Brunauer-Emmett-Teller (BET) analysis (BSD INSTRUMENT BSD-PM1/2, Beijing, China) of Spent FCC catalyst.

Testing Content	BET Surface Area (m^2^/g)	Matrix Surface Area (m^2^/g)	Micropore Surface Area (m^2^/g)	Total Pore Volume (cc/g)	Micropore Volume (cc/g)
Before the experiment	129	60	69	0.154	0.031
After the experiment	104	52	52	0.128	0.025

**Table 6:** Fitting results of activation energy.

	Inner Liner					Tread Rubber				
W_t_/%	0	10	20	30	40	0	10	20	30	40
E/kJ·mol^−1^	288	232.3	214.7	209.1	203.7	238.9	201.8	188.8	191.3	184.2
Fitting function	Y = a/(X + b) + c									
Fitting result	Y = 8.058/(X + 0.0776) + 187					Y = 3.873/(X + 0.06267) + 177.1				
R^2^	0.99					0.98				

Note: W_t_—The weight ratios of catalyst to rubber material.

**Table 7:** Detailed experimental conditions.

No. of Experiment	Rubber	W_R_/mg	Catalyst	W_C_/mg	T_H_/°C	*E*/kJ·mol^−1^
1	Inner liner	15.44				288.03
2		15.34	I	4.60		209.19
3		15.67	I	4.64	1000	287.03
4		15.54	II	4.59		257.39
5		15.11	II	4.58	1000	279.7

Note: W_R_—Average weight of rubber, W_C_—Average weight of catalyst, T_H_—Heating temperature of the catalyst, I—Unused catalysts, II—Used catalysts.

## Data Availability

Data sharing not applicable.

## References

[1] Gars J., Olovsson C. (2019). Fuel for economic growth?. J. Econ. Theory.

[2] Xu F., Wang B., Yang D., Ming X., Jiang Y., Hao J., Qiao Y. (2018). TG-FTIR and Py-GC / MS study on pyrolysis mechanism and products distribution of waste bicycle tire. Energy Convers. Manag..

[3] Thomas B.S., Gupta R.C. (2016). A comprehensive review on the applications of waste tire rubber in cement concrete. Renew. Sustain. Energy Rev..

[4] Koskin A.P., Zibareva I.V., Vedyagin A.A., Nanda S., N. Vo D.-V., Sarangi P.K. (2020). Conversion of Rice Husk and Nutshells into Gaseous, Liquid, and Solid Biofuels. Biorefinery of Alternative Resources: Targeting Green Fuels and Platform Chemicals.

[5] Gao J., Zhang Y., Meng D., Jiao T., Qin X., Bai G., Liang P. (2019). Effect of ash and dolomite on the migration of sulfur from coal pyrolysis volatiles. J. Anal. Appl. Pyrolysis.

[6] Xu F., Wang B., Yang D., Hao J., Qiao Y., Tian Y. (2018). Thermal degradation of typical plastics under high heating rate conditions by TG-FTIR: Pyrolysis behaviors and kinetic analysis. Energy Convers. Manag..

[7] Wu M., Zhao M., Chang G., Hu X., Guo Q. (2019). A composite obtained from waste automotive plastics and sugarcane skin flour: Mechanical properties and thermo-chemical analysis. Powder Technol..

[8] Guo Q., Yue X., Wang M., Liu Y. (2010). Pyrolysis of scrap printed circuit board plastic particles in a fluidized bed. Powder Technol..

[9] Cao X., Li L., Shitao Y., Liu S., Hailong Y., Qiong W., Ragauskas A.J. (2019). Catalytic conversion of waste cooking oils for the production of liquid hydrocarbon biofuels using in-situ coating metal oxide on SBA-15 as heterogeneous catalyst. J. Anal. Appl. Pyrolysis.

[10] Li L., Yan B., Li H., Yu S., Liu S., Yu H., Ge X. (2018). SO42−/ZrO2 as catalyst for upgrading of pyrolysis oil by esterification. Fuel.

[11] Li L., Ding Z., Li K., Xu J., Liu F., Liu S., Yu S., Xie C., Ge X. (2016). Liquid hydrocarbon fuels from catalytic cracking of waste cooking oils using ultrastable zeolite USY as catalyst. J. Anal. Appl. Pyrolysis.

[12] Li L., Quan K., Xu J., Liu F., Liu S., Yu S., Xie C., Zhang B., Ge X. (2014). Liquid hydrocarbon fuels from catalytic cracking of rubber seed oil using USY as catalyst. Fuel.

[13] Zhong W., Guo Q., Wang X., Zhang L. (2013). Catalytic hydroprocessing of fast pyrolysis bio-oil from Chlorella. J. Fuel Chem. Technol..

[14] Qi P., Chang G., Wang H., Zhang X., Guo Q. (2018). Production of aromatic hydrocarbons by catalytic co-pyrolysis of microalgae and polypropylene using HZSM-5. J. Anal. Appl. Pyrolysis.

[15] Williams P.T. (2013). Pyrolysis of waste tyres: A review. Waste Manag..

[16] Doğan O., Celik M.B., Özdalyan B. (2012). The effect of tire derived fuel/diesel fuel blends utilization on diesel engine performance and emissions. Fuel.

[17] Wang K., Xu Y., Duan P., Wang F., Xu Z. (2019). Thermo-chemical conversion of scrap tire waste to produce gasoline fuel. Waste Manag..

[18] Elbaba I.F., Williams P.T. (2013). High yield hydrogen from the pyrolysis–catalytic gasification of waste tyres with a nickel/dolomite catalyst. Fuel.

[19] İlkılıç C., Aydın H. (2011). Fuel production from waste vehicle tires by catalytic pyrolysis and its application in a diesel engine. Fuel Process. Technol..

[20] Yuwapornpanit R., Jitkarnka S. (2015). Cu-doped catalysts and their impacts on tire-derived oil and sulfur removal. J. Anal. Appl. Pyrolysis.

[21] Muenpol S., Jitkarnka S. (2016). Effects of Fe supported on zeolites on structures of hydrocarbon compounds and petrochemicals in waste tire-derived pyrolysis oils. J. Anal. Appl. Pyrolysis.

[22] Kordoghli S., Khiari B., Paraschiv M., Zagrouba F., Tazerout M. (2019). Production of hydrogen and hydrogen-rich syngas during thermal catalytic supported cracking of waste tyres in a bench-scale fixed bed reactor. Int. J. Hydrogen Energy.

[23] Li Y., Paranthaman M.P., Akato K., Naskar A.K., Levine A.M., Lee R.J., Kim S.-O., Zhang J., Dai S., Manthiram A. (2016). Tire-derived carbon composite anodes for sodium-ion batteries. J. Power Sources.

[24] Williams P.T., Brindle A.J. (2002). Catalytic pyrolysis of tyres: Influence of catalyst temperature. Fuel.

[25] Li L., Quan K., Xu J., Liu F., Liu S., Yu S., Xie C., Ge X. (2014). Preparation of basic mesoporous molecular sieves K_2_O/Mg-MCM-41 and its catalytic performance on the cracking of soybean oils. J. Anal. Appl. Pyrolysis.

[26] Zhang X., Li C., Tian A., Guo Q., Huang K. (2019). Influence of CaO and HZSM-5 on oxygen migration in Chlorella vulgaris polysaccharide pyrolysis. Carbon Resour. Convers..

[27] Xie C., Liu F., Yu S., Xie F., Li L., Zhang S., Yang J. (2008). Catalytic cracking of polypropylene into liquid hydrocarbons over Zr and Mo modified MCM-41 mesoporous molecular sieve. Catal. Commun..

[28] Li L., Yan B., Li H., Yu S., Ge X. (2020). Decreasing the acid value of pyrolysis oil via esterification using ZrO2/SBA-15 as a solid acid catalyst. Renew. Energy.

[29] Wu C., Williams P.T. (2010). Pyrolysis–gasification of post-consumer municipal solid plastic waste for hydrogen production. Int. J. Hydrogen Energy.

[30] Namchot W., Jitkarnka S. (2016). Catalytic pyrolysis of waste tire using HY/MCM-41 core-shell composite. J. Anal. Appl. Pyrolysis.

[31] Elbaba I.F., Wu C., Williams P.T. (2011). Hydrogen production from the pyrolysis–gasification of waste tyres with a nickel/cerium catalyst. Int. J. Hydrogen Energy.

[32] Czajczyńska D., Krzyżyńska R., Jouhara H., Spencer N. (2017). Use of pyrolytic gas from waste tire as a fuel: A review. Energy.

[33] Wang S., (Max) Lu G.Q. (1998). Role of CeO2 in Ni/CeO2–Al2O3 catalysts for carbon dioxide reforming of methane. Appl. Catal. B Environ..

[34] Rodríguez E., Félix G., Ancheyta J., Trejo F. (2018). Modeling of hydrotreating catalyst deactivation for heavy oil hydrocarbons. Fuel.

[35] Marafi M., Stanislaus A., Furimsky E. (2017). Handbook of Spent Hydroprocessing Catalysts: Second Edition. Handb. Spent Hydroprocessing Catal. Second Ed..

[36] Pathak A., Srichandan H., Kim D.J. (2019). Column bioleaching of metals from refinery spent catalyst by Acidithiobacillus thiooxidans: Effect of operational modifications on metal extraction, metal precipitation, and bacterial attachment. J. Environ. Manage..

[37] Wei X., Zhong H., Yang Q., Yao E., Zhang Y., Zou H. (2019). Studying the mechanisms of natural rubber pyrolysis gas generation using RMD simulations and TG-FTIR experiments. Energy Convers. Manag..

[38] Grieco E., Bernardi M., Baldi G. (2008). Styrene-butadiene rubber pyrolysis: Products, kinetics, modelling. J. Anal. Appl. Pyrolysis.

[39] Cao L., Sinha T.K., Tao L., Li H., Zong C., Kim J.K. (2019). Synergistic reinforcement of silanized silica-graphene oxide hybrid in natural rubber for tire-tread fabrication: A latex based facile approach. Compos. Part B Eng..

[40] Zhang X., Cai L., Wang C., He A. (2019). Data on the evolution of curing characteristics and properties during the room-temperature annealing process in SSBR/BR gums and SSBR/BR/SiO2 composites. Data Br..

[41] Kordoghli S., Paraschiv M., Kuncser R., Tazerout M., Zagrouba F. (2017). Catalysts’ influence on thermochemical decomposition of waste tires. Environ. Prog. Sustain. Energy.

[42] Demirbas A. (2004). Determination of calorific values of bio-chars and pyro-oils from pyrolysis of beech trunkbarks. J. Anal. Appl. Pyrolysis.

[43] Bridgwater A.V. (2003). Renewable fuels and chemicals by thermal processing of biomass. Chem. Eng. J..

[44] Hilal DemirbaŞ A. (2005). Yields and heating values of liquids and chars from spruce trunkbark pyrolysis. Energy Sources.

[45] Fernandez-Lopez M., Pedrosa-Castro G.J., Valverde J.L., Sanchez-Silva L. (2016). Kinetic analysis of manure pyrolysis and combustion processes. Waste Manag..

[46] Starink M.J. (1996). A new method for the derivation of activation energies from experiments performed at constant heating rate. Thermochim. Acta.

[47] Wang C., Zhao B., Tian X., Wang K., Tian Z., Han W., Bian H. (2020). Study on the pyrolysis kinetics and mechanisms of the tread compounds of silica-filled discarded car tires. Polymers.

[48] Yang S.J., Chen Y.W. (1995). Chiuping-Li The interaction of vanadium and nickel in USY zeolite. Zeolites.

[49] Matar S., Hatch L.F. (2001). Synthetic Petroleum-Based Polymers. Chem. Petrochem. Processes.

[50] Kan T., Strezov V., Evans T. (2017). Fuel production from pyrolysis of natural and synthetic rubbers. Fuel.

[51] Thakar N., Schildhauer T.J., Buijs W., Kapteijn F., Moulijn J.A. (2007). Evaluation of deactivation mechanisms of Pd-catalyzed hydrogenation of 4-isobutylacetophenone. J. Catal..

[52] Boxiong S., Chunfei W., Cai L., Binbin G., Rui W. (2007). Pyrolysis of waste tyres: The influence of USY catalyst/tyre ratio on products. J. Anal. Appl. Pyrolysis.

[53] Shen B., Wu C., Wang R., Guo B., Liang C. (2006). Pyrolysis of scrap tyres with zeolite USY. J. Hazard. Mater..

[54] Nishikawa J., Nakamura K., Asadullah M., Miyazawa T., Kunimori K., Tomishige K. (2008). Catalytic performance of Ni/CeO_2_/Al_2_O_3_ modified with noble metals in steam gasification of biomass. Catal. Today.

[55] Mortola V.B., Damyanova S., Zanchet D., Bueno J.M.C. (2011). Surface and structural features of Pt/CeO2-La2O3-Al2O3 catalysts for partial oxidation and steam reforming of methane. Appl. Catal. B Environ..

[56] Le T., Wang Q., Ravindra A.V., Li X., Ju S. (2019). Microwave intensified synthesis of Zeolite-Y from spent FCC catalyst after acid activation. J. Alloys Compd..

[57] Domingues C., Correia M.J.N., Carvalho R., Henriques C., Bordado J., Dias A.P.S. (2013). Vanadium phosphate catalysts for biodiesel production from acid industrial by-products. J. Biotechnol..

[58] El-Akkad T.M., Khalil A.M., Attia G., Nashed S. (1982). Surface and structural properties of 3, 5 and 10A synthetic zeolites. Thermochim. Acta.

[59] Tian H., Huang C., Fan Z., Spivey J.J., Roberts G.W., Davis B.H.B.T.-S. (2001). Metals on a Novel USY Zeolite after Hydrothermal Aging. Catalyst Deactivation 2001.

